# Training set optimization is a feasible alternative for perennial orphan crop domestication and germplasm management: an *Acrocomia aculeata* example

**DOI:** 10.3389/fpls.2024.1441683

**Published:** 2024-09-10

**Authors:** Evellyn G. O. Couto, Saulo F. S. Chaves, Kaio Olimpio G. Dias, Jonathan A. Morales-Marroquín, Alessandro Alves-Pereira, Sérgio Yoshimitsu Motoike, Carlos Augusto Colombo, Maria Imaculada Zucchi

**Affiliations:** ^1^ Deparment of Agronomy, Federal University of Viçosa, Viçosa, Brazil; ^2^ Deparment of General Biology, Federal University of Viçosa, Viçosa, Brazil; ^3^ Genetics and Molecular Biology Department, Biology Institute, University of Campinas (UNICAMP), Campinas, Brazil; ^4^ Research Center of Plant Genetic Resources, Campinas Agronomic Institute, Campinas, Brazil; ^5^ Department of Genetics, "Luiz de Queiroz" College of Agriculture, University of São Paulo, Piracicaba, Brazil

**Keywords:** genomic prediction, macauba, perennial native species, risk-averse decisions, GBLUP, BayesB

## Abstract

Orphan perennial native species are gaining importance as sustainability in agriculture becomes crucial to mitigate climate change. Nevertheless, issues related to the undomesticated status and lack of improved germplasm impede the evolution of formal agricultural initiatives. *Acrocomia aculeata* - *a* neotropical palm with potential for oil production - is an example. Breeding efforts can aid the species to reach its full potential and increase market competitiveness. Here, we present genomic information and training set optimization as alternatives to boost orphan perennial native species breeding using *Acrocomia aculeata* as an example. Furthermore, we compared three SNP calling methods and, for the first time, presented the prediction accuracies of three yield-related traits. We collected data for two years from 201 wild individuals. These trees were genotyped, and three references were used for SNP calling: the oil palm genome, *de novo* sequencing, and the *A. aculeata* transcriptome. The traits analyzed were fruit dry mass (FDM), pulp dry mass (PDM), and pulp oil content (OC). We compared the predictive ability of GBLUP and BayesB models in cross- and real validation procedures. Afterwards, we tested several optimization criteria regarding consistency and the ability to provide the optimized training set that yielded less risk in both targeted and untargeted scenarios. Using the oil palm genome as a reference and GBLUP models had better results for the genomic prediction of FDM, OC, and PDM (prediction accuracies of 0.46, 0.45, and 0.39, respectively). Using the criteria PEV, r-score and core collection methodology provides risk-averse decisions. Training set optimization is an alternative to improve decision-making while leveraging genomic information as a cost-saving tool to accelerate plant domestication and breeding. The optimized training set can be used as a reference for the characterization of native species populations, aiding in decisions involving germplasm collection and construction of breeding populations

## 1 Introduction

As the world increasingly emphasizes sustainability in agricultural ecosystems amid climate change, the exploration of orphan native species gains importance. These species represent novel sources of germplasm, holding alleles that confer resistance to both biotic and abiotic stresses. They are adapted to local environmental conditions and can thrive under less intensive, more sustainable agricultural management practices (Ulian et al., 2020; Yaqoob et al., 2023). *Acrocomia aculeata* (Jacq.) Lood. ex Mart (Arecaceae), 2n = 2x = 30, a neotropical native palm, stands out as an economically promising orphan species. This palm is considered the most widespread in Brazil, occurring in all Brazilian biomes but the Pampa, in the Southern region. Particularly, Cerrado is the biome in which it most occurs (Lima et al., 2018; Scariot et al., 1995; Lorenzi, 2010). It demonstrates resilience to severe drought and exhibits wide adaptation across the Brazilian territory, being able to recolonize devastated areas, with high solar incidence and low water index (Lima et al., 2018; Cardoso et al., 2017; Vargas-Carpintero et al., 2021). Several studies emphasize the *A. aculeata* fruits as a valuable source of nutrients, finding applications in the food, cosmetic, pharmaceutical, and biofuel industries (Lescano et al., 2021; Aguieiras et al., 2014; Monteiro-Alfredo et al., 2023). Nowadays, there is an increasing interest in the *A. aculeata* fruits due to their high yield and oil quality derived from pulp and kernel (Evaristo et al., 2016; Lanes et al., 2016; Madeira et al., 2024). In making a parallel with its relative, oil palm (*Elaeis guineensis*), *A. aculeata* is more adapted as it grows in regions where the oil palm could not due to insufficient water availability (Pires et al., 2013). In fact, *A. aculeata* is a native pioneer species and, different from oil palm, *A. aculeata* plantations can be installed without touching rainforests and protected biomes.

Despite the research highlighting its potential, formal agricultural initiatives involving *A. aculeata* remain scarce. The limited use of *A. aculeata* can be attributed to several factors, such as i) undomesticated status, meaning that optimal agronomic practices particular to each environmental condition have not been firmly established; ii) lack of improved germplasm and operational issues, which increases the risk of agricultural losses and hampers field management due to the lack of uniformity in the field; and iii) post-harvesting challenges, i.e. issues such as low processing efficiency and the absence of a well-established industry to absorb the production (Resende et al., 2020; Cardoso et al., 2017; Vargas-Carpintero et al., 2022).

Usage of genomic information can potentially expedite the domestication process of *A. aculeata* and advance the development of improved cultivars. Moreover, it can enhance the precision of breeding efforts, optimizing both time and resources—financial and human (Laviola et al., 2022). For instance, genomic information can be leveraged in genomic selection/prediction models (Bernardo, 1994; Meuwissen et al., 2001), which, after training a statistical model enriched with phenotypic and genomic information, allows for the selection of candidates based only on its allelic constitution. However, comprehensive research detailing how to leverage genomic selection in the species’ breeding program effectively remains scarce. Currently, most *A. aculeata* applied researches are focused on entering the pre-breeding stage (Vargas-Carpintero et al., 2021). These initial steps include the characterization and collection of wild individuals, the establishment of germplasm banks, and the formation of breeding populations (Vargas-Carpintero et al., 2021; Lanes et al., 2016). These initial steps would be simple and straightforward had not the perennial nature of *A. aculeata*: under natural conditions, it begins producing fruits in the fourth or fifth year of life (Forest Resources Development Branch, 1986), making each step time-consuming. This trend extends beyond *A. aculeata* to encompass other orphan perennial native species. Given this context, breeders need to explore alternative applications for genomic selection/prediction beyond the conventional approach employed in established breeding populations (Tanaka and Iwata, 2018; Gorjanc et al., 2016). This exploration can facilitate the selection of plants with phenotypic traits of commercial interest, thereby accelerating their domestication. The standard procedure usually involves training a genomic prediction model using the entire population as a training set to predict the performance of future genetically related populations (Grattapaglia, 2022; Crossa et al., 2017).

**Figure 1:**
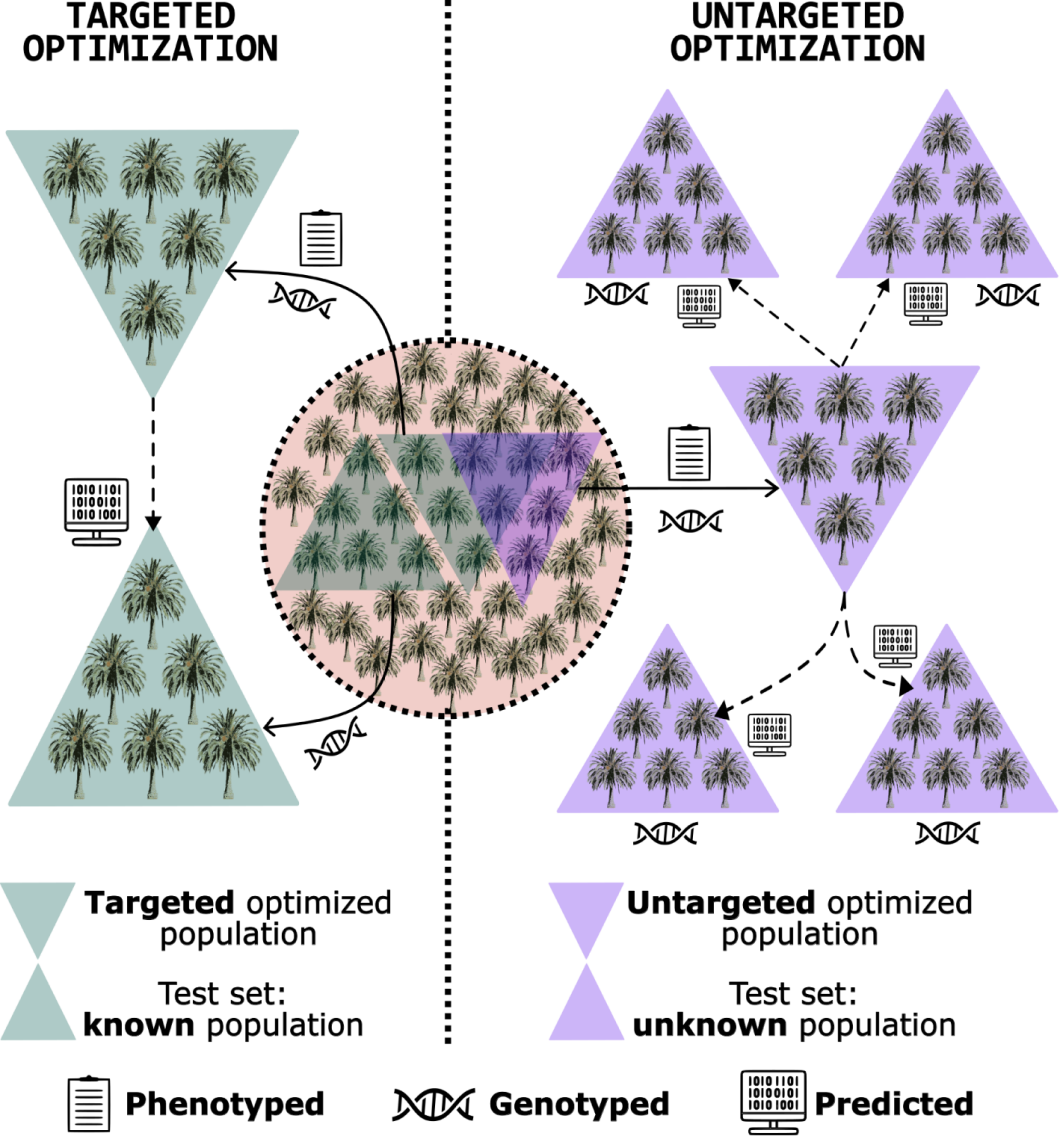
Schematic illustration presenting the fundamental concept of targeted and untargeted training set optimization: a genotyped and phenotyped population is utilized to identify a subset of individuals for enhanced genomic prediction accuracy. Two scenarios are illustrated: i) optimization directed at predicting the performance of a known, unphenotyped population (targeted optimization); and ii) optimization to predict the performance of an unknown, unphenotyped population (untargeted optimization). Despite being distinct objectives, the same individuals can be chosen to form optimized training sets in both situations.

An alternative is the training set optimization. This method was developed to select a subset of genotypes from the population that shares the closest relationship with the test set, providing more reliable predictions (Rincent et al., 2012; Akdemir et al., 2015). This approach can reduce phenotyping costs, as only genotypes within the optimized training set are phenotyped. Moreover, it enhances prediction efficiency, as the relationship between training and test sets significantly influences genomic prediction accuracy (Bustos-Korts et al., 2016; Isidro et al., 2015). In the pre-breeding context, the training set optimization can be employed to characterize germplasm, guiding decisions on the collection of accessions and utilization of specific genotypes as parents (Yu et al., 2016; Akdemir and Sánchez, 2016). This approach can steer the breeding program toward an efficient and sustainable trajectory from its inception. Training set optimization is categorized into two scenarios (**Figure 1**) (Isidro y Sánchez and Akdemir, 2021; Fernández-González et al., 2023):

- Targeted optimization: involves leveraging a known, genotyped population to refine the composition of a training set, aiming to identify a subset of phenotyped and genotyped individuals that closely resemble those in the testing set, who are also known. This optimized training set is then employed to predict the performance of the genotypes within the testing set. Atanda et al. (2021) and Roth et al. (2020) showed this strategy’s efficiency in improving genomic predictions in maize and apple, respectively. In the realm of pre-breeding for perennial native species, targeted optimization offers a strategic approach to conserving resources in germplasm phenotyping. By selecting a subset of individuals for both phenotyping and genotyping, this method identifies the most promising candidates to predict the performance of the remaining individuals - which would be only genotyped, thereby streamlining the process and reducing costs.
- Untargeted optimization: entails selecting a subset of phenotyped and genotyped candidates that effectively capture the available diversity within the dataset, without necessarily requiring knowledge of the testing set. The objective is to create an optimized training robust enough to be a reliable tool for predicting the performance of any genetically related unphenotyped population. The strategies used by Yu et al. (2016) in sorghum (*Sorghum* spp.) and Rio et al. (2019) in maize (when they built a diverse training set, with representatives of multiple groups) are categorized in this class. In the context of pre-breeding for perennial native species, untargeted optimization serves as a valuable tool for guiding accession collection strategies. By genotyping a subset of candidates within a specific area, this approach facilitates the identification and collection of individuals that best represent the genetic diversity of the region. Leveraging the principle that geographically proximate trees are more likely to be genetically related than those further apart, the sampled subset offers a comprehensive representation of the local genetic pool and is well-suited for predicting the performance of individuals within that area.

In this study, we illustrate the effective utilization of genomic selection in both predicting the performance of unphenotyped trees and optimizing training sets for recurrent objectives in perennial orphan native species, taking *A. aculeata* breeding as an example. Our objectives were i) to assess the predictive ability of different statistical-genetics models and SNP calling methods for predicting *A. aculeata* fruit productive traits; and ii) to show the application of training set optimization to select a subset of wild individuals that more accurately captures the diversity within a population and, or are the most genetically close to a known, unphenotyped population. We posit that the methodology exemplified in this study regarding training set optimization serves as a cost-effective alternative for characterizing native species populations - not only for *A. aculeata*, selecting germplasm for gene banks, and making risk-averse decisions.

## 2 Materials and methods

### 2.1 Plant material

**Figure 2:**
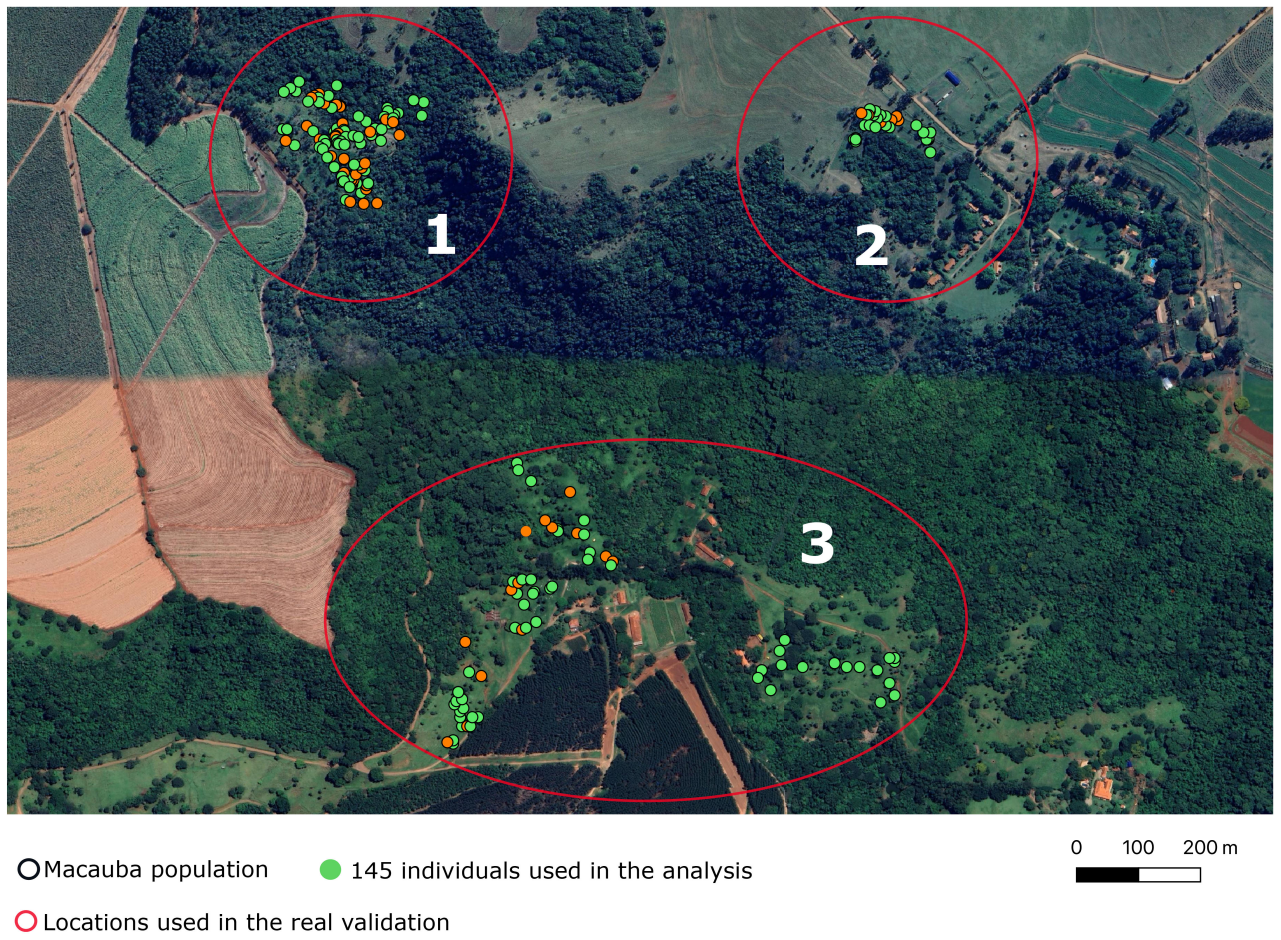
Map of the areas where wild *Acrocomia aculeata* populations were sampled. Each dot represents an individual. The green ones were used in the subsequent analyses. The red circles and the numbers within them represent the locations used in the real validation.

Two hundred and one individuals of *Acrocomia aculeata* were sampled from three rural areas in Dourado, a city in São Paulo state, Brazil. We prioritized palm trees that had ripe fruits in the year 2019/2020, and evaluated these trees in the years 2019/2020 and 2021/2022. We collected data of 201 palm trees in three rural areas (henceforth referred as “locations”) approximately 500 meters apart (**Figure 2**). The sampled individuals were part of wild populations, and data was collected *in loco*. In other words, there is no specific experimental design. The collected phenotypes relate to vegetative growth and yield-related traits. In this study, we considered three of them: fruit dry mass (FDM), pulp dry mass (PDM), and pulp oil content (OC). During data collection, fruits were carefully dissected and separated into four components: husk, pulp, endocarp, and almond. FDM and PDM measurements were obtained after drying the samples in a ventilated oven at 36°C, while OC was assessed based on the dry mesocarp mass using Near-infrared Spectroscopy. For more detailed information, refer to Couto et al. (2024).

### 2.2 Genotypic information

Genotyping was conducted using the genotyping-by-sequencing protocol (GBS). Genomic DNA was isolated from leaf material following the methodology described by Doyle and Doyle (1990). For GBS library preparation, two restriction enzymes, *NsiI* and *MseI* (New England Biolabs), were employed according to the protocol outlined by Poland et al. (2012), with modifications as per Díaz et al. (2021). The 201 sample libraries were sequenced in a single run on an Illumina HiSeq3000 platform, configured with single-end and 101bp settings. Following quality control and demultiplexing of the sequencing reads, SNP calling was performed using three strategies due to the absence of a reference genome for *A. aculeata*: utilizing the genome of *Elaeis guineensis* EG5 (NCBI GCA_000442705.1), the transcriptome of *A. aculeata* (Bazzo et al., 2018), and the *de novo* pipeline (Stacks v.1.42, Catchen et al., 2011). SNPs were filtered based on the following criteria: maximum number of alleles = 2, minor allele frequency ≥ 0.01, sequencing depth ≥ 3X, mapping quality ≥ 20, maximum percentage of 30% missing data per locus, and 45% missing data per individual. This filtering process resulted in the identification of a total of 10,444 SNPs in 158 individuals using the oil palm reference genome, 4,329 SNPs in 167 individuals using the transcriptome reference, and 27,410 SNPs in 153 individuals from the *de novo* pipeline. Missing data were imputed using the Beagle 5.3 software (Browning et al., 2021). Further details on the genotyping sequencing methodology are found in Couto et al. (2024).

### 2.3 Genomic selection/prediction models

To perform the analyses described in this section, we kept data from trees that had information from the tree SNP calling methods, and no missing data regarding the three analysed traits. Thus, from the 201 available plants, 145 were kept. The letter V (v= 1,2,… V) will represent this amount in the mathematical notations below.

First, we built the genomic relationship matrices $\left(G_{x}\right)$ using the R package AGHmatrix (Amadeu et al., 2023). We utilized the equation proposed by VanRaden (2008):


(1) $$G_{x}=\frac{{\overset{˘}{M}}_{x}{\overset{˘}{M}}_{x}^{'}}{2\sum_{j}p_{j}\left(1-p_{j}\right)}$$

where ${\overset{˘}{M}}_{x}=M_{x}-2P_{x}$ , in which $M_{x}$ is the SNP matrix obtained using the $x^{th}$ SNP-calling reference, $P_{x}$ is a matrix of frequencies for the alternative allele in each locus, and $p_{j}$ is the alternative allele frequency in the $j^{th}$ locus. $G_{x}$ are V×V matrices. We investigated the genetic diversity among plants using $G_{x}$ in a principal component analysis (PCA), using the R package FactoMineR (Lê et al., 2008).

Next, we extracted the adjusted means of each trait using the following model:


(2) $$y_{t}=1\mu +X_{1}a+X_{2}g+\epsilon$$

where $y_{t}$ is the N×1 vector of phenotypic records of the $t^{th}$ trait, in which N is the number of records; μ is the intercept, connected to $y_{t}$ by a N×1 vector of ones (**1**); a and g are the M×1 and V×1 vectors of fixed effects of years ( m∈{1,2},M=2 ) and genotypes, followed by their N×M and N×V incidence matrices, respectively; and ϵ is the N×1 vector of residual effects [ $\epsilon ∼\text{N}\left(0,\sigma_{\epsilon }^{2}I_{N}\right)$ , where $\sigma_{\epsilon }^{2}$ is the residual variance and $I_{N}$ is an identity matrix of order N ]. These means were used to train the genomic selection/prediction models.

#### 2.3.1 GBLUP

We used the following GBLUP (Bernardo, 1994) model:


(3) $${\bar{y}}_{t}=1\mu +Zg+\epsilon$$

where ${\bar{y}}_{t}$ is the V×1 vector of adjusted means, and g is the V×1 vector of random genetic effects [ $g∼\text{N}\left(0,\sigma_{g}^{2}G_{x}\right)$ , where $\sigma_{g}^{2}$ is the genetic variance], accompanied by its V×V incidence matrix. The other terms were previously declared in Equation 2. Note that the model described in Equation 3 was fitted thrice, each time with a different $G_{x}$ , i.e., a genomic kinship matrix originated from markers from different SNP calling methods. The variance component estimates of each model were used to calculate the narrow-sense heritabilities of each trait ( $h_{t_{x}}^{2}$ ):


(4) $$h_{t_{x}}^{2}=\frac{\sigma_{g_{x}}^{2}}{\sigma_{g_{x}}^{2}+\sigma_{\epsilon }^{2}}$$

we computed the approximate standard error of these estimates using the Delta method [see Holland et al. (2002) for more details about this method].

The model of Equation 2 and the GBLUP model of Equation 3 were fitted using the ASReml-R package, version 4.2.0.267 (The VSNi Team, 2023).

#### 2.3.2 BayesB

We fitted the following BayesB model:


(5) $${\bar{\text{y}}}_{t}=1\mu +M_{x}\beta +\epsilon$$

where ***β*** is the vector of random marker effects. We used the default priors of the R package we employed to fit the model, BGLR (Pérez and de los Campos, 2014).

#### 2.3.3 Cross- and real validation

To evaluate the predictive prowess of the models, we implemented a *k*-fold cross-validation approach. The dataset was partitioned into five folds (*k* = 5), with one fold excluded (20%) in each round to be predicted by the remaining four (80%). Each fold had 29 individuals. We iterated this process five times to mitigate bias linked to fold composition, randomly shuffling the fold makeup with each repetition. At the end of each iteration, we integrated the outputs in a single V×1 vector ( $\hat{y}$ ). We computed the correlation between predicted and observed values ( $\rho_{\bar{y}\hat{y}}$ ) and the mean squared prediction error ( $MSPE=\frac{1}{V}\sum_{v=1}^{V}{\left(\bar{y}-\hat{y}\right)}^{2}$ ).

We estimate the real predictive accuracy leveraging the empirical grouping based on the geographic distance between trees. This was done employing a leave-one-out scheme. In each iteration, we used data from two locations (say, 1 and 3) to predict the values of the third location (e.g., location 2). The number of individuals per location was 66, 24 and 55 (locations 1, 2, and 3, respectively). In this validation, we also computed $\rho_{\bar{y}\hat{y}}$ and MSPE .

### 2.4 Training set optimization

After determining the SNP-calling reference yielding the best prediction results, we employed the corresponding SNP matrix, $M_{x}$ , and genomic relationship matrix, $G_{x}$ , in training set optimization algorithms. Optimization was carried out in two primary scenarios: targeted and untargeted (**Figure 1**). In both scenarios, we used the memetic evolutionary algorithm implemented in the R package TrainSel (Akdemir et al., 2021) and the genetic algorithm of the TSDFGSR package (Ou and Liao, 2019) to perform the recursive search of optimized training sets. The fundamental concept involves testing various genotype combinations to construct the training set and utilizing an optimization criterion to assess set quality. These algorithms might yield different outcomes based on factors such as the initial training set composition and the number of iterations. To overcome this issue, we performed the recursive search 50 times. We then selected the genotypes most frequently included in the optimized training set across these repetitions. To monitor convergence, we examined the progress of the best training set optimization criterion value across iterations in each repetition (an example is provided in **Supplementary Figure S1** in the **Supplementary Material**). For a deeper understanding of the memetic evolutionary and genetic algorithms, refer to Holland (1992), Hart et al. (2005); Akdemir et al. (2015) and Akdemir et al. (2021). The subsequent sections provide a detailed breakdown of the procedures and methods used to ascertain the composition of the optimized training set in each scenario. To differ from the notation adopted to represent the total number of genotypes, the training set size will be represented by $\overset{˘}{V}$ and the test set size will be $\ddot{V}(\ddot{V}=V-\overset{⌣}{V})$

#### 2.4.1 Untargeted optimization

This scenario was subdivided according to the training set size. We tested two sizes: 50 and 100 genotypes. We used six optimization criteria to determine the best training set composition, namely:

- D-optimality (Wald, 1943): The idea is to maximize the log-determinant of **S**, the $\overset{˘}{V}\times \overset{˘}{V}$ matrix of principal components derived from the centered SNP matrix. In this context, maximizing |**SS**'| is equivalent to minimizing the variance of marker effects. Using **S** instead of $M_{x}$ increases computational efficiency (Ou and Liao, 2019; Akdemir et al., 2015).
- CDmean (Laloë, 1993): This metric is taken from the mean of the diagonal values of the coefficient of determination (CD) matrix, given by:

(6) $$\left(GZ^{\prime }PZG\right)⊘G$$

in which P is the projection matrix [ $P=V^{-1}-V^{-1}1\left(1^{\prime }V^{-1}1\right)1^{\prime }V^{-1}$ where $V=ZG{\text{Z}}^{\prime }+R$ , with R being the residual covariance matrix] and ⊘ is the element-wise division. The closer the CDmean to 1, the better.

- CDmin (Akdemir et al., 2021): It has the structure of the previously described CDmean, but instead of taking the mean, CDmin takes the minimum value of the CD matrix diagonal elements.
- PEV (Akdemir et al., 2015): An ideal training set minimizes the prediction error variance (PEV) in the testing set. In the untargeted case, all candidates who were not part of the candidate training set composed the testing set. Leveraging this division, we can partition S into $S_{tr}$ , related to the $\overset{˘}{V}$ genotypes that compose the candidate training set, and $S_{ts}$ , which contains information of the $\ddot{V}$ remaining genotypes. Thence:

(7) $$PEV\approx \left(1_{\ddot{V}},S_{ts}\right){\left[{\left(1_{\overset{˘}{V}},S_{tr}\right)}^{'}\left(1_{\overset{˘}{V}},S_{tr}\right)+\text{λ}I_{\left(J-1\right)}\right)}^{-1}{\left(1_{\ddot{V}},S_{ts}\right)}^{'}$$

where **1** is a vector of ones and **I** is an identity matrix, whose sizes are indicated by their subscript. *λ* is a regularization parameter, fixed in /J1 , with *J* being the number of markers.

- r-score (Ou and Liao, 2019): This criterion is based on the correlation between genomic-estimated breeding values and phenotypic values in a test set. The r-score is obtained as follows:

(8) $$\text{r}-\text{score}=\frac{q_{12}}{\sqrt{q_{1}q_{2}}}$$

where $q_{12}=Tr\left[S_{ts}^{'}\left(I_{\ddot{V}}-{\bar{J}}_{\ddot{V}}\right)S_{ts}AS_{tr}\right]$ , $q_{1}=\left(\ddot{V}-1\right)+Tr\left[S_{ts}\left(I_{\ddot{V}}-{\bar{J}}_{\ddot{V}}\right)S_{ts}\right]$ , and $q_{2}=Tr\left[A^{\prime }S_{ts}^{'}\left(I_{\ddot{V}}-{\bar{J}}_{\ddot{V}}\right)S_{ts}A]+Tr[A^{\prime }S_{tr}^{'}\left(I_{\ddot{V}}-{\bar{J}}_{\ddot{V}}\right)S_{tr}A\right]$ . ${\bar{J}}_{\ddot{V}}$ is a $\ddot{V}\times \ddot{V}$ matrix filled with /V¨1 , and $A=S_{tr}^{'}{\left(S_{tr}S_{tr}^{'}+\text{λ}I_{V}\right)}^{-1}$ , with λ being a regularization parameter ( λ=1 for the r-score). Like a regular correlation, the higher the r-score, the better.

- MaxiMin (Johnson et al., 1990): The sole non-parametric criterion tested in the untargeted scenario, it aims to maximize the minimum genetic distance among the training set components.

We assessed the performance of optimized training sets generated by each optimization criterion through a cross-validation procedure, akin to the one detailed in section 2.3.3. To substantiate our hypothesis that employing an optimized training set yields lower risk compared to random sampling from the population, we conducted 100 cross-validations using random training sets. Instead of using a *k*-fold structure, we held the training population size constant at 50 or 100.

#### 2.4.2 Targeted optimization

We adopted the same framework as the real validation outlined in section 2.3.3 to optimize a targeted training set. In this context, we maintained the training set size at 50 genotypes and explored two scenarios: predicting group 3 using groups 1 and 2 and predicting group 2 using groups 1 and 3. Four of the six optimization criteria utilized in the untargeted optimization—CDmean, CDmin, PEV, and r-score—were also employed here. The distinction lies in the fact that the testing set is confined to genotypes exclusively from a single group. Alongside these criteria, we introduced two additional ones:

- MiniMax (Johnson et al., 1990): the idea is to minimize the maximum genetic distance between genotypes of the training and test sets.
- Multiple design criterion (Akdemir and Sánchez, 2016): Blending the goals of untargeted and targeted optimizations, this criterion emphasizes training sets with the maximum mean genetic distance within the set and the minimum mean genetic distance between the training set and the test set. For this criterion, in particular, the selection of optimized training sets was confined to a predefined empirical range of acceptable distance values (refer to **Supplementary Figure S2** in the **Supplementary Material**).

After identifying the optimized training sets, we evaluated their predictive ability using the leave-one-out scheme previously described (section 2.3.3). Following what was done in the untargeted scenario, we also evaluated the efficiency of 100 training sets composed of randomly sampled genotypes.

### 2.5 Core collection

Some optimization criteria have the same objective as defining a core collection: to select a subset of individuals that better represents genetically the whole population. Here, we evaluate the Entry-to-nearest-entry (E-NE) method, implemented in the corehunter R package (De Beukelaer et al., 2018), as a seventh alternative to defining an optimized training set, in both untargeted and targeted scenarios. E-NE’s background is the genetic distances based on molecular data. The algorithm can yield highly diversified sets since it considers the average distance between each selected individual and the closest other candidate. We used the Modified Roger’s as a distance measure, given by (Thachuk et al., 2009):


(9) $$0\leq M\;R_{vv^{\prime }}=\frac{1}{2J}\sqrt{\sum_{j=1}^{J}\sum_{a=1}^{A}{\left(p_{vja}-p_{v^{\prime }ja}\right)}^{2}}\leq 1$$

where $MR_{vv^{\prime }}$ is the Modified Roger’s distance between individual *v* and *v* ^′^, *A* is the number of alleles per locus, and *p* is the relative frequency of allele *a*.

We also computed the expected proportion of heterozygous loci per individual (*HE*) and the coverage of alleles in the core collection (*CV*), given by, respectively (Thachuk et al., 2009):


(10) $$0\leq H\;E=\frac{1}{J}\sum_{j=1}^{J}\left(1-\sum_{a=1}^{A}p_{ja}^{2}\right)\leq 1$$

(11) $$CV=\left(1-\frac{{\text{Ξ}}_{core}}{{\text{Ξ}}_{pop}}\right)*100$$

where ${\text{Ξ}}_{core}$ and ${\text{Ξ}}_{pop}$ are the set of alleles found in the core collection and the population, respectively.

In the untargeted scenario, all individuals were candidates to be part of the core collection. We followed the adopted variation in the sample size (50 and 100). In the targeted scenario, only genotypes from the non-excluded group were considered to form the core collection.

All analyses were performed in the R software environment, version 4.3.2 (R Core Team, 2023). We build all plots using features of the tidyverse library, with add-ins from the packages gghighlight, ggpubr and ggpattern (Wickham et al., 2019; Yutani, 2023; Kassambara, 2023; FC et al., 2024).

## 3 Results

**Figure 3:**
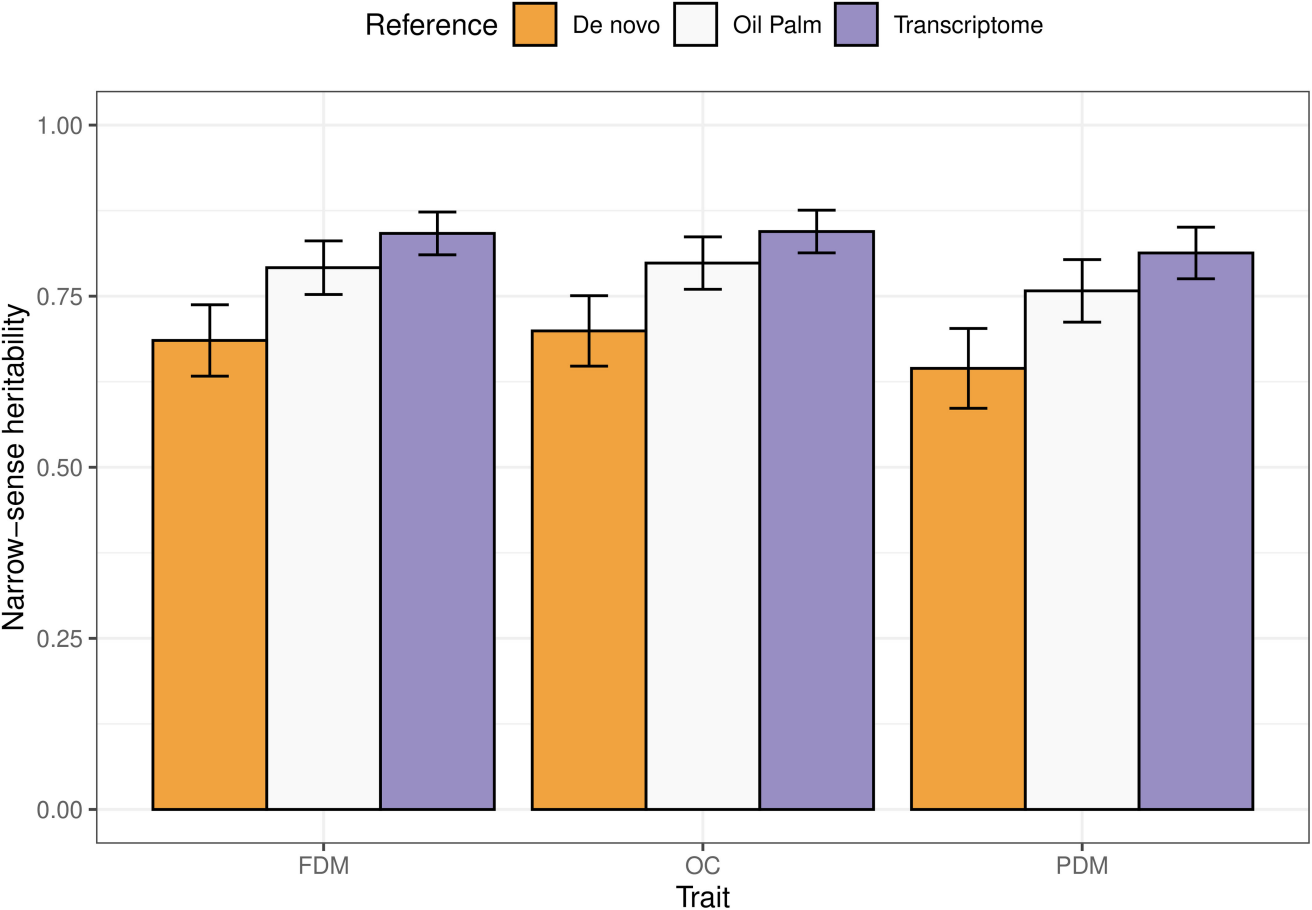
Barplots representing the narrow-sense heritabilities estimated for each trait (FDM, fruit dry mass; OC, oil content, and PDM, pulp dry mass), using different references for SNP calling. The error bars illustrate the upper and lower limits taking an approximate standard error calculated using the Delta method.

All results varied according to the reference genome used for SNP calling. The narrow-sense heritabilities ranged from 0.68 to 0.84, 0.7 to 0.85 and 0.64 to 0.81 for FDM, OC and PDM, respectively (**Figure 3**). A clear pattern is observed: using the *A. aculeata* transcriptome as reference always yielded the highest heritability values, followed by the oil palm reference genome and the *de novo* sequencing.

### 3.1 Genomic prediction models

**Figure 4:**
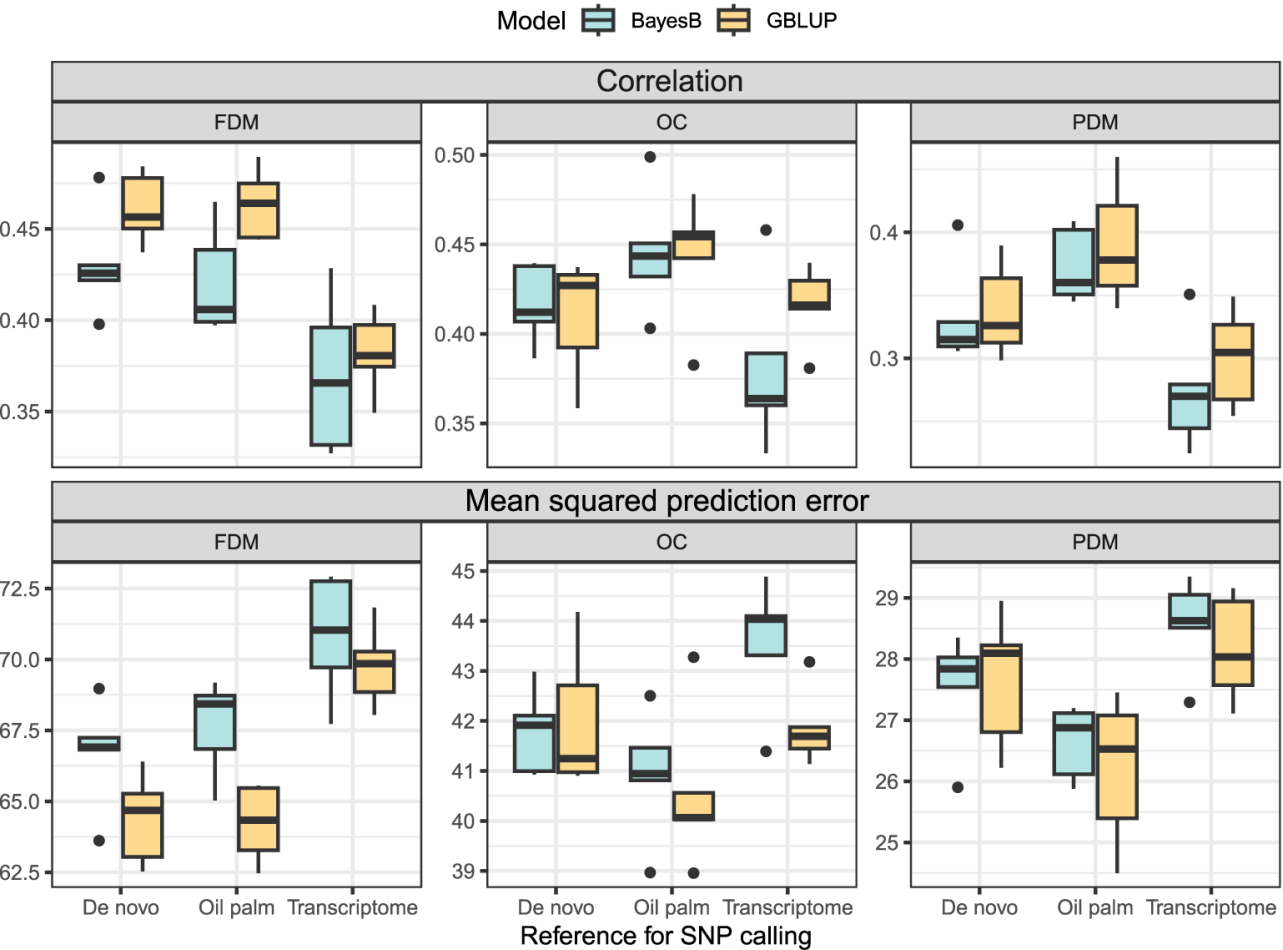
Boxplots depict the cross-validation results of the GBLUP and the BayesB models for each trait (FDM, fruit dry mass; OC, oil content, and PDM, pulp dry mass) and SNP calling reference (*De novo* sequencing, oil palm genome and *A. aculeata* transcriptome). The top three plots show the correlation between observed and predicted values, and the lower three plots display the mean squared prediction error.

**Figure 5:**
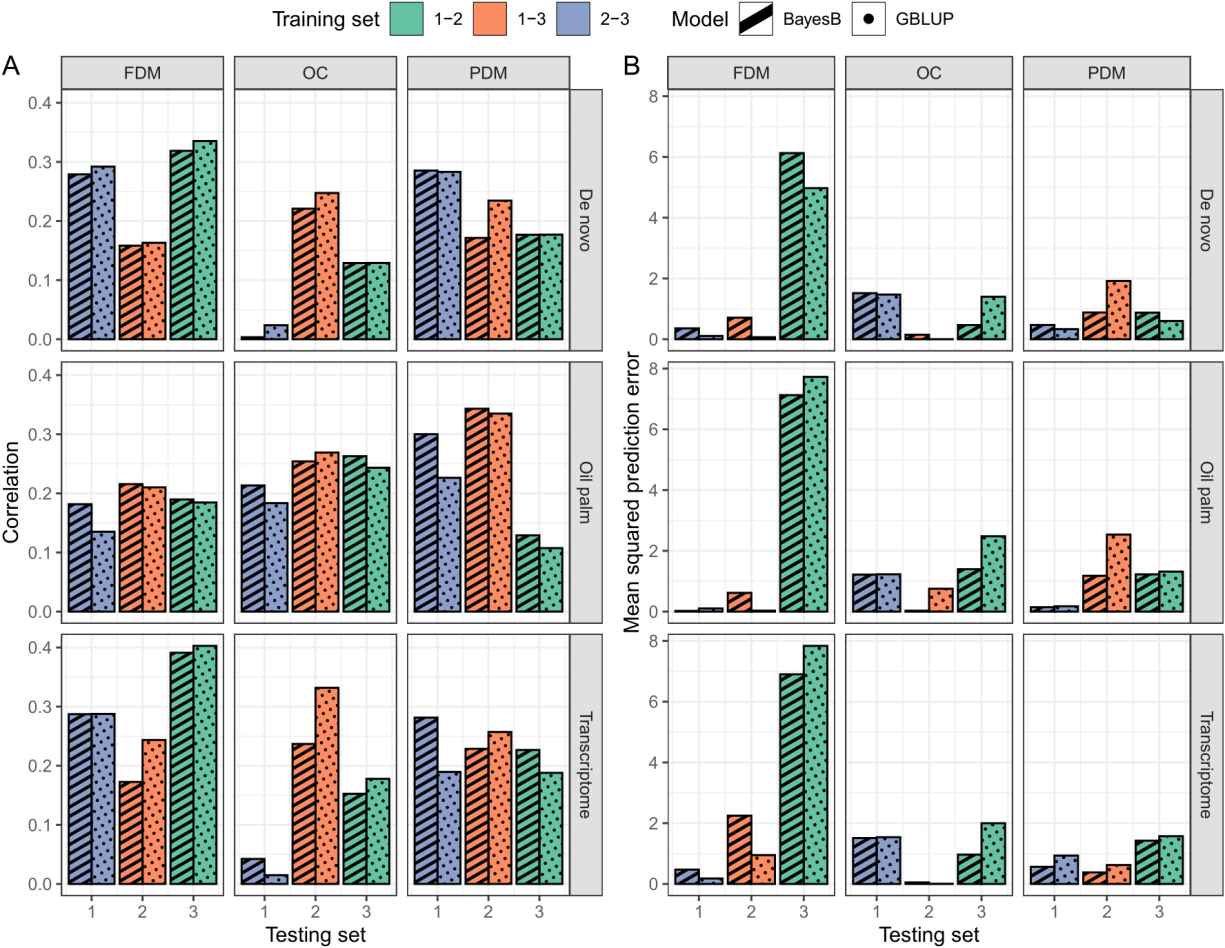
Real validation outcomes per trait (columns, FDM, fruit dry mass; OC, oil content, and PDM, pulp dry mass) and SNP calling reference (rows, *De novo* sequencing, oil palm genome and *A. aculeata* transcriptome) of the GBLUP (dotted pattern) and BayesB (striped pattern) models. In Plot **(A)**, the correlation between observed and predicted values is presented, while Plot **(B)** shows the mean squared prediction error. Bar colors correspond to the groups constituting the training set, with the *x*-axis indicating the test set group.

In the cross-validation, oil content (OC) and fruit dry mass (FDM) exhibited similar $\rho_{\bar{y}\hat{y}}$ , but OC demonstrated a lower *MSPE*. Pulp dry mass (PDM) achieved the lowest *MSPE*, although $\rho_{\bar{y}\hat{y}}$ was inferior to the other two traits (**Figure 4**). In the real validation, the results were highly variable, depending on the reference for SNP calling, the model used and the training/test set division (**Figure 5**). Overall, using groups 1 and 3 to predict group 2 seemed more successful for OC. In FDM, predicting group 3 using groups 1 and 2 yielded the highest $\rho_{\bar{y}\hat{y}}$ on two out of three occasions (**Figure 5A**), but also had the highest *MSPE* (**Figure 5B**). For PDM, the models more efficiently predicted group 1 using groups 2 and 3.

The utilization of the oil palm reference genome as a reference for SNP calling yielded superior prediction results in the cross-validation (**Figure 4**). Overall, the GBLUP model demonstrated higher $\rho_{\bar{y}\hat{y}}$ and lower *MSPE* than the BayesB model across most traits and SNP calling methods (**Figure 4**). Due to the variation in the real validation, we considered only the cross-validation results to determine that using GBLUP and considering the oil palm genome as a reference for SNP calling is more adequate for *A. aculeata* genomics when breeding for the studied traits. These findings can inform future decisions related to genomic management in *A. aculeata*. Considering these outcomes, we chose to proceed with training set optimization using only the oil palm reference genome and GBLUP for cross-validation.

### 3.2 Untargeted training set optimization

**Figure 6:**
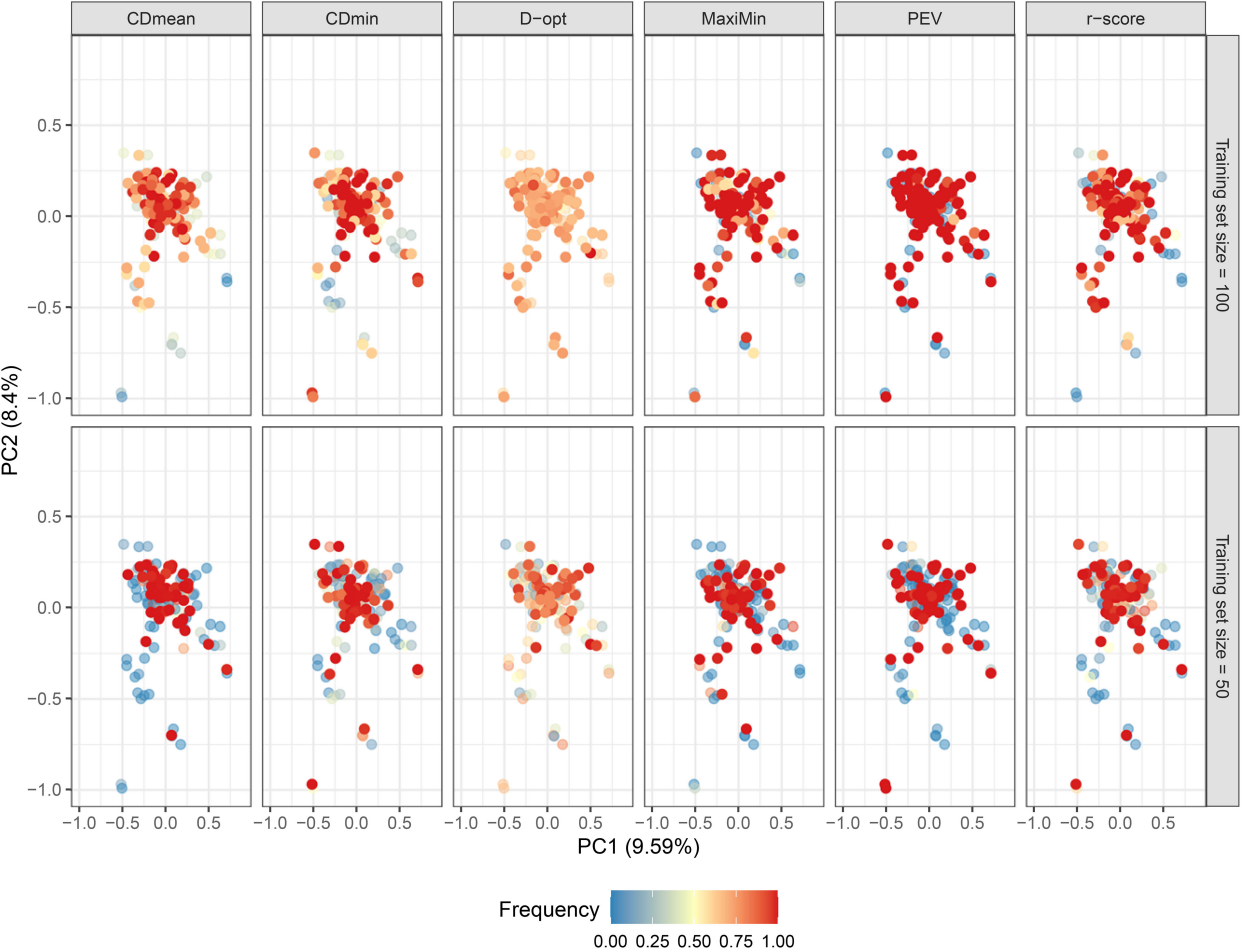
Biplot depicting the genotype distribution using the first two principal components. Dots are colored according to their frequency in the untargeted optimized training set after 50 runs, following various optimization criteria. Opaque and intensely colored dots indicate the 100 (upper plots) and 50 (lower plots) genotypes selected for the optimized training set.

The studied population lacks a distinct structure, and most genotypes exhibit a close genetic relationship, irrespective of geographic distance (**Figures 6**; **Supplementary Figure S3** of the **Supplementary Material**). This condition theoretically enhances genomic prediction and training set optimization. As anticipated, the composition of the untargeted optimal training set varied based on the optimization criterion. **Figure 6** illustrates how genotypes frequently present in the optimized training sets across runs are dispersed in the PCA biplot in all criteria. This underscores the objective of untargeted optimization: selecting a subset of genotypes that can effectively represent the genomic diversity in the population. The PEV criterion exhibited the highest consistency in selecting the same genotypes across runs, while D-optimality was the least consistent (**Figure 6**; **Supplementary Figure S4** of the **Supplementary Material**).

**Figure 7:**
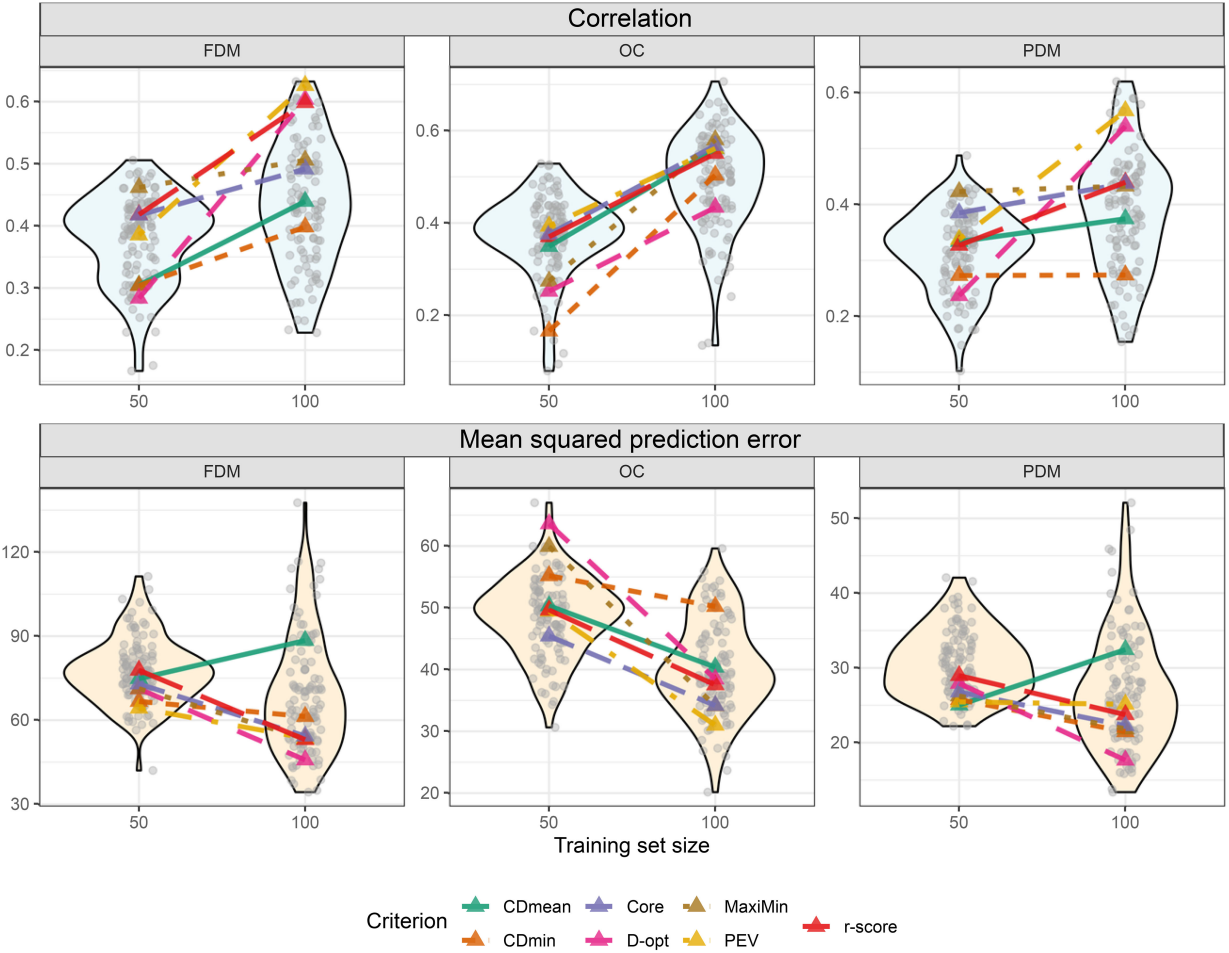
Violin plot illustrating cross-validation outcomes post untargeted training set optimization for two different training set sizes (y-axis). Transparent grey circles depict results from 100 cross-validations using randomly sampled training sets. Colored triangles represent optimized training sets based on different optimization criteria. The upper plots display the correlation between observed and predicted values, while the three lower plots show the mean squared prediction error.

No optimization criterion consistently outperformed others in all situations during cross-validation. The criterion performance varied depending on the trait and training set size. For instance, considering FDM, the MaxiMin criterion demonstrated the highest $\rho_{\bar{y}\hat{y}}$ and the lowest *MSPE* with a training set size of 50. However, with a size of 100, it ranked fourth in $\rho_{\bar{y}\hat{y}}$ and fourth lowest in *MSPE* (**Figure 7**). Still, two criteria stand out for always yielding good results: PEV and r-score. Another interesting outcome is the high performance of the training sets composed of the core collection, which always featured amongst the top three criteria. Notably, some random-sampled training sets outperformed all optimized training sets (**Figure 7**). This is important to stress that the objective of the optimization is not to find the training set for the highest $\rho_{\bar{y}\hat{y}}$ and lowest *MSPE* but to provide sets with lower risk in predictions, prioritizing risk-averse decisions. In the untargeted scenario, this is achieved using PEV, r-score, or the core collection.

### 3.3 Targeted training set optimization

**Figure 8:**
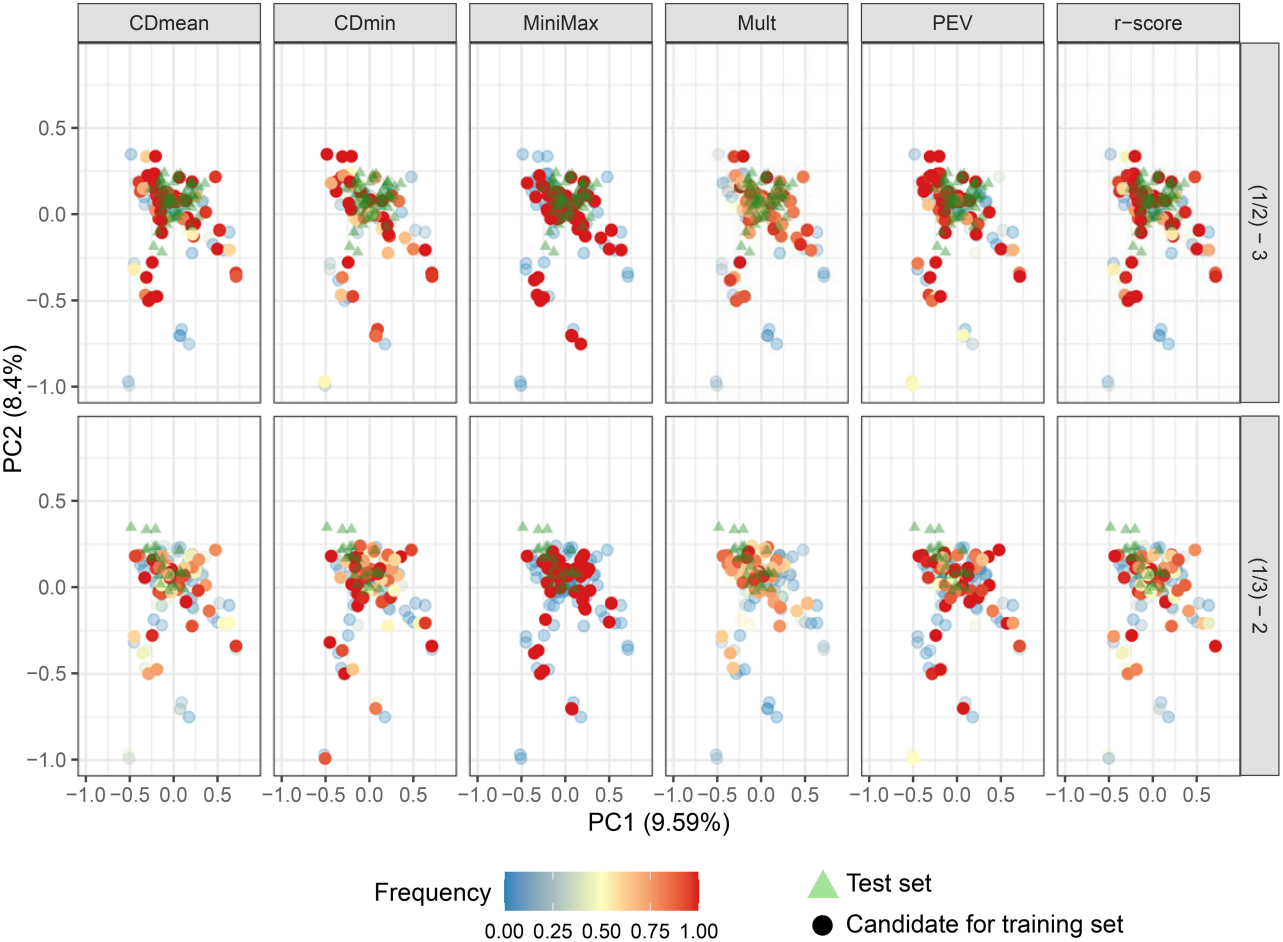
Biplot depicting the genotype distribution in the first two principal components. Triangles represent the genotypes in the test set, and circle the candidates to compose the training set. Circle colors indicate the frequency of their inclusion in the targeted optimized training set across 50 runs with different optimization criteria. Intensely colored, opaque dots represent the 50 genotypes chosen for the optimized training set when the test set was group 3 (upper plots) and group 2 (lower plots).

When predicting group 3, all criteria except CDmin and Mult consistently selected the same representatives, indicating close genetic relation to the test set. Minimax and PEV showed more stable performance across runs when group 2 was the test set (**Figures 8**; **Supplementary Figure S5** of the **Supplementary Figure S4**). Convergence issues impeded the prediction of group 1 using groups 2 and 3.

**Figure 9:**
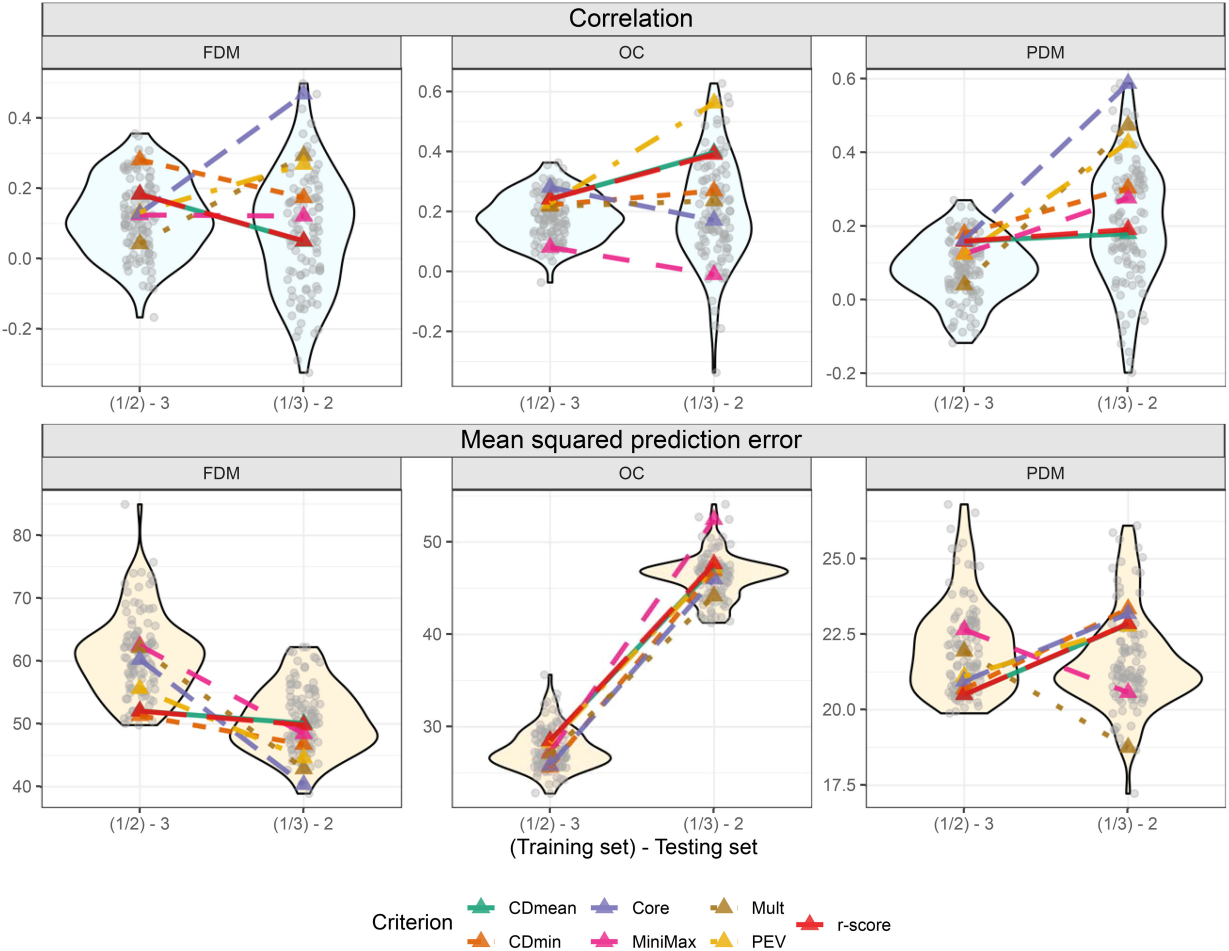
Violin plot illustrating cross-validation outcomes after targeted training set optimization, with two training set sizes on the *x*-axis. Grey transparent circles depict results from the 100 cross-validations using randomly sampled training sets. Colored triangles represent optimized training sets based on different optimization criteria. The upper plots display the correlation between observed and predicted values, while the three lower plots show the mean squared prediction error.

In the cross-validations, the targeted training set performances varied more among optimization criteria compared to the untargeted scenario (**Figure 9**). Using FDM as an example again, CDmin provided the best-optimized training set when group 3 was the test set, but had only the fourth-best performance when group 2 was the test set. It is challenging to identify standout optimization criteria in this scenario. However, it is noteworthy that all criteria, except MiniMax for OC, yielded results above average in all situations. Furthermore, like in the untargeted optimization, using the core collection is an interesting alternative for the targeted scenario. This method produced good results for OC when group 3 was the test set and for FDM and PDM, where it provided the training set with the best outcome among all tested sets, including random-sampled sets (**Figure 9**).

## 4 Discussion

In this study, we underscored the considerable impact of the reference genome on genomic-related outcomes. Optimal results in the studied traits were attained by leveraging the oil palm as the reference genome in conjunction with the GBLUP statistical-genetic model. Our investigation established the viability of training set optimization in the pre-breeding context as a robust strategy for ensuring reliable predictions, both in untargeted and targeted scenarios. Additionally, we highlighted the efficacy of utilizing the core collection, demonstrating its capacity to yield high-performance results in prediction models. These findings instill confidence in breeders of native orphan species, providing a secure foundation for genomic-based decisions in crucial breeding activities like germplasm characterization and breeding population structuring.

### 4.1 Genomic prediction models

Depending on the genetic architecture of the trait, GBLUP may yield suboptimal results, as it assumes equal variances for all markers. On the other hand, BayesB assumes that some markers are not in linkage disequilibrium with the quantitative trait loci (QTL), and do not segregate (i.e., have nil variance) (Meuwissen et al., 2001). Still, the BayesB priors do not reflect the real genetic architecture of the studied traits, as the general assumptions of GBLUP provided the best prediction outcomes in the cross-validation.

The importance of a high-quality reference genome in ensuring accurate predictions has been well-established in genomic research (Benevenuto et al., 2019). Given the absence of a dedicated reference genome for *A. aculeata*, our study demonstrated that employing the reference genome of a closely related species (*E. guineensis*, Lopes et al., 2018) resulted in the highest predictive performances across the tested models. This result makes sense, since *E. guineensis* and *A. aculeate* are phylogenetically close species (Francisconi et al., 2023). Notably, previous studies utilizing genome-wide SNP markers in similar contexts have employed different references. For instance, Díaz et al. (2021) relied on *de novo* sequencing to study genetic diversity in the *Acrocomia* genus, and Couto et al. (2024) considered multiple references in their comprehensive genome-wide association study (GWAS). In a formal breeding pipeline, it is not feasible to test several SNP-calling methods, per objective, per trait. Thus, based on our results and in the absence of a proper reference genome for *A. aculeata*, we recommend the usage of *E. guineensis* reference genome as an alternative.

Focusing on the results of the oil palm reference genome, the narrow-sense heritabilities for the studied traits hovered around 0.75. Such values serve as an upper limit for prediction accuracy and play a crucial role as benchmarks for evaluating the models’ predictive capabilities. It is worth noting that the values are close to what was previously found for the same traits in another population (Costa et al., 2018). While heritability values can vary across populations, having this reference value is pivotal for guiding decision-making in subsequent studies. Another important observation is that cross-validation tends to bias upward the real predictive ability of models (Gezan et al., 2017), as shown by the difference between the outcomes of cross- and real validations. The training set composition and its relation to the test set is one of the causes of this pattern since predictive ability in real validations varied per trait and groups used as training and test sets. This justifies the concern of seeking an optimized training set.

### 4.2 Training set optimization

The composition of the optimized training set introduces an element of uncertainty, which we sought to mitigate by employing multiple iterations of the recursive search (Akdemir et al., 2015). This inherent uncertainty is counterbalanced by the consistently above-average performance exhibited by most of the optimized training sets across various optimization criteria (Isidro et al., 2015; Fernández-González et al., 2023). Our study emphasizes that training sets selected through the PEV and r-score criteria, along with the utilization of the core collection, consistently enhance the predictive capacity of the GBLUP model in all scenarios. Therefore, it is reasonable to endorse these criteria for *A. aculeata* in situations where cross-validation may not be feasible. It is important to note that the efficacy of optimized training sets, based on different criteria, is contingent on factors such as training set size, trait architecture, and population structure (Isidro et al., 2015; Rincent et al., 2012; Ou and Liao, 2019). Consequently, thorough validation is crucial before implementing this strategy in other species or even in different populations of the same species (Tanaka and Iwata, 2018; Akdemir et al., 2015; Guo et al., 2019).

Training set optimization emerges as a viable strategy for harnessing genomic information, particularly in the pre-breeding stages. While studies by Yu et al. (2016) and Tanaka and Iwata (2018) have demonstrated this in the context of large genebanks for staple crops, our proposal extends this approach to perennial species, drawing inspiration from the dataset employed in this study: wild individuals assessed over multiple years across distinct areas. Perennial species require extended evaluation periods to draw reliable conclusions. Training set optimization can facilitate germplasm characterization by concentrating phenotyping efforts on a subset of representative individuals, reducing the associated costs. Moreover, employing training set optimization allows for investigating whether the performance of trees in a less accessible area can be predicted from a more accessible one. In scenarios where germplasm collection is the objective, the genotypes within the optimized training set are likely to represent a diverse sample of alleles present in the entire population. For genebank characterization and breeding population structuring, crossings between components of the optimized training set offer the potential to generate progenies with alleles from different sources, providing a promising foundation for recurrent selection programs. Another important detail is that the extrapolation of results based on the optimized training set is only as reliable as the sample quality. For instance, in this study, it would be inappropriate to assert that the diversity captured by the optimized training set represents the entire *A. aculeata* diversity in Brazil. To achieve this, we would need to sample individuals from populations occurring in other biomes, altitudes, soil classes, etc. [See, for example, Resende et al. (2020)]. This is a topic for future studies.

It is essential to note that the methodology outlined in this study does not negate the significance of other information sources, such as a plant’s performance for a specific trait, its reproductive capacity, or the impacts on genetic diversity and inbreeding (Simiqueli et al., 2018; Díaz-Hernández et al., 2024). Instead, it should be regarded as an additional tool to mitigate the risk of erroneous decisions in the initial stages of breeding programs, thus safeguarding subsequent results. This integrated approach aims to enhance the efficiency and reliability of breeding efforts in perennial species.

### 4.3 Perspectives

The results of this study could further be refined under conditions mirroring a formal genebank setting—where trees share the same age and are arranged in a controlled experimental design. Enhancements in reliability could be achieved through additional measures such as expanding the sampled population size, increasing the number of measurements, evaluating multiple environments, and accounting for genotype-by-environment interactions. These considerations collectively underscore the adaptability and utility of the optimization strategies in a variety of breeding program scenarios for orphan species. Genomic information stands as a powerful tool poised to greatly enhance the efficiency of breeding programs, especially as the costs associated with sequencing continue to decline. This is particularly pertinent for orphan species, which are gaining prominence in response to the growing need to broaden the genetic and nutritional foundations of crops. Many of these species find themselves in the pre-breeding stage, often constrained by limited human and financial resources. The optimization strategies demonstrated in this study present a practical and cost-effective means to harness the potential benefits of genomic information.

## 5 Concluding remarks

The training set optimization methods exhibited in this study are alternatives to decrease the risk of making a flawed decision while leveraging genomic information as a cost-saving tool. We showed that the predictive ability of genomic prediction models is hardly below average when using an optimized training set. This provides breeders with the reliability required to use the optimized training set as a reference for the characterization of native species populations, aiding in decisions involving germplasm collection and construction of breeding populations. Particularly for *A. aculeata*, we showed that an appropriate genome reference is vital for SNP calling, and consequently, any initiative that involves genomic information. While waiting for a proper reference genome, using the oil palm genome as a reference for SNP calling seems to yield better results for the genomic prediction of FDM, OC and PDM. By using this reference for SNP calling in GBLUP models, we reached mean prediction accuracies of 0.46, 0.45 and 0.39 for FDM, OC and PDM, respectively, in cross-validations. Genomic prediction proved to be feasible and would represent a boost in efficiency if adopted in the breeding program pipeline, given the species’ perennial nature.

## Data Availability

Publicly available datasets were analyzed in this study. This data can be found here: https://github.com/evellyngocouto/Macauba_GWAS.
