# Supplementary material for: Training set optimization is a feasible alternative for perennial orphan crop domestication and germplasm management: an Acrocomia aculeata example
Source: Front Plant Sci. 2024 Sep 10;15:1441683. doi: 10.3389/fpls.2024.1441683 (PMC11423296; doi:10.3389/fpls.2024.1441683)
Supplement: Supplementary file 1 [file DataSheet1.pdf]

Supplementary Material

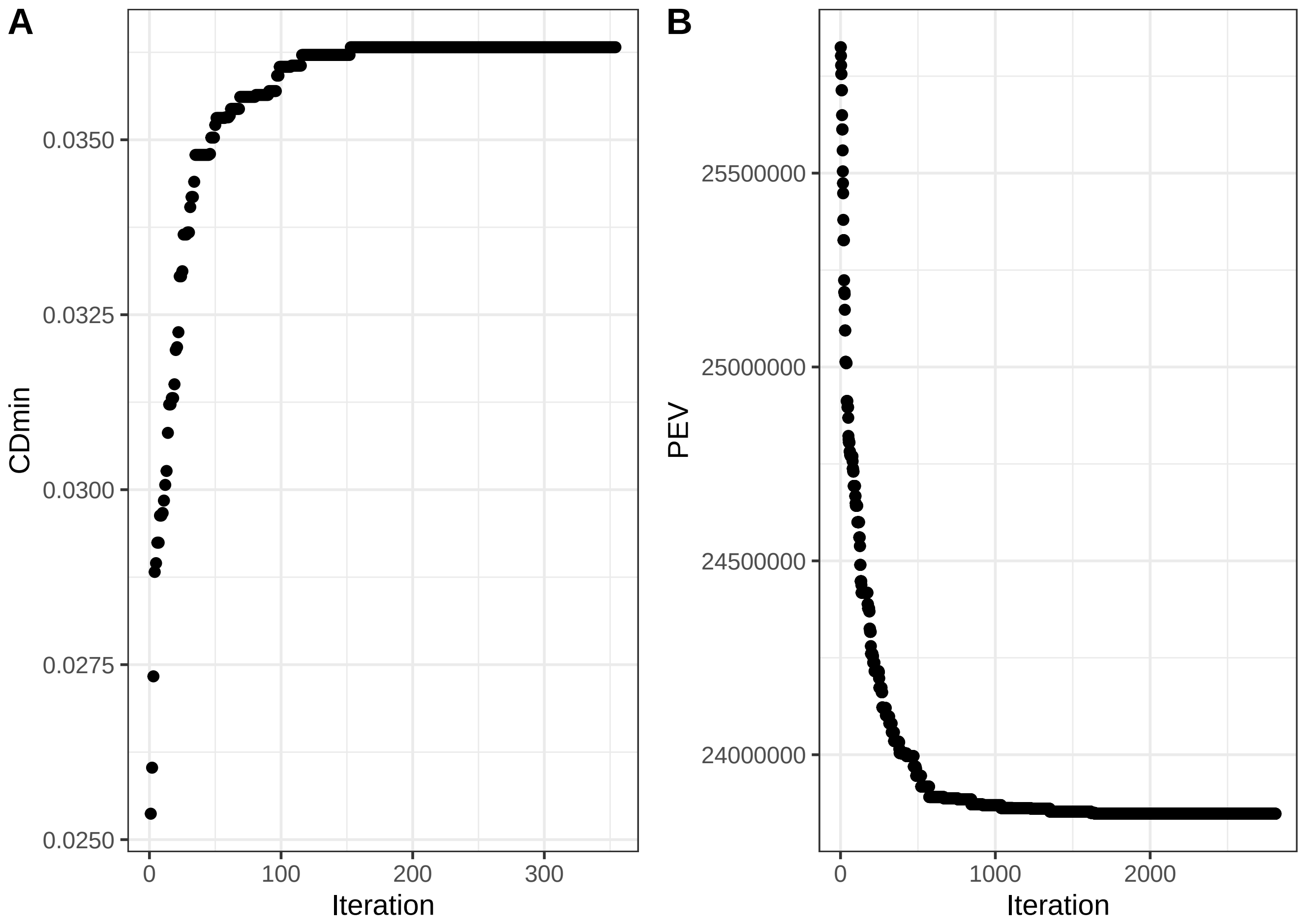

Figure S1. Example of plots used to check the convergence of each repetition in the recursive search: CDmin (**A**) using the package `TrainSel`; and PEV (**B**), using the package `TSDFGS`.

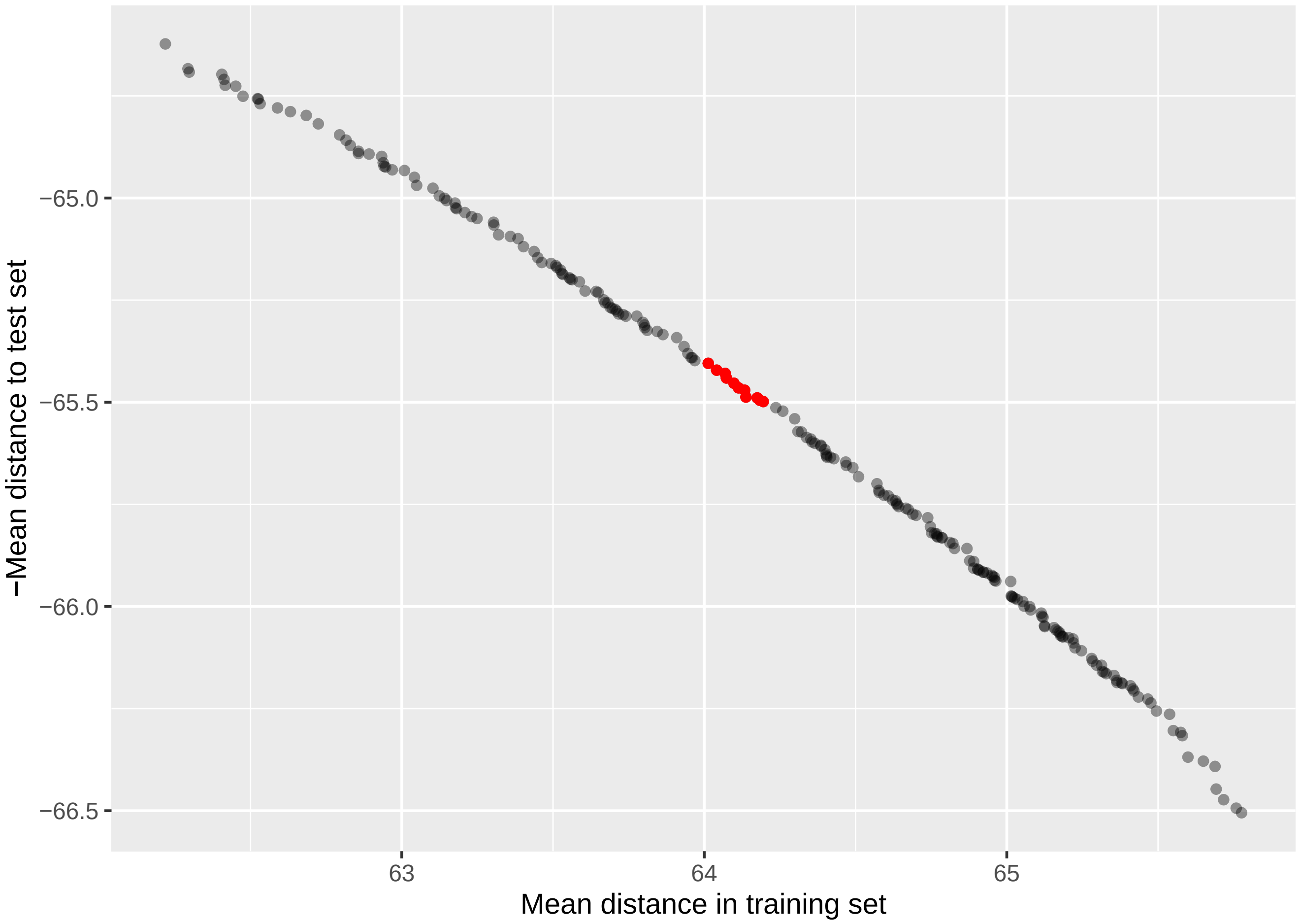

Figure S2. Plot used to empirically define the range of acceptable distance values in the multiple design criterion. In the example, we adopted a minimum mean distance within the training equal to 64 and a maximum mean distance to the test set of 65.5. Each dot represents a possible training set, and highlighted dots are the ones that fit the established criteria.

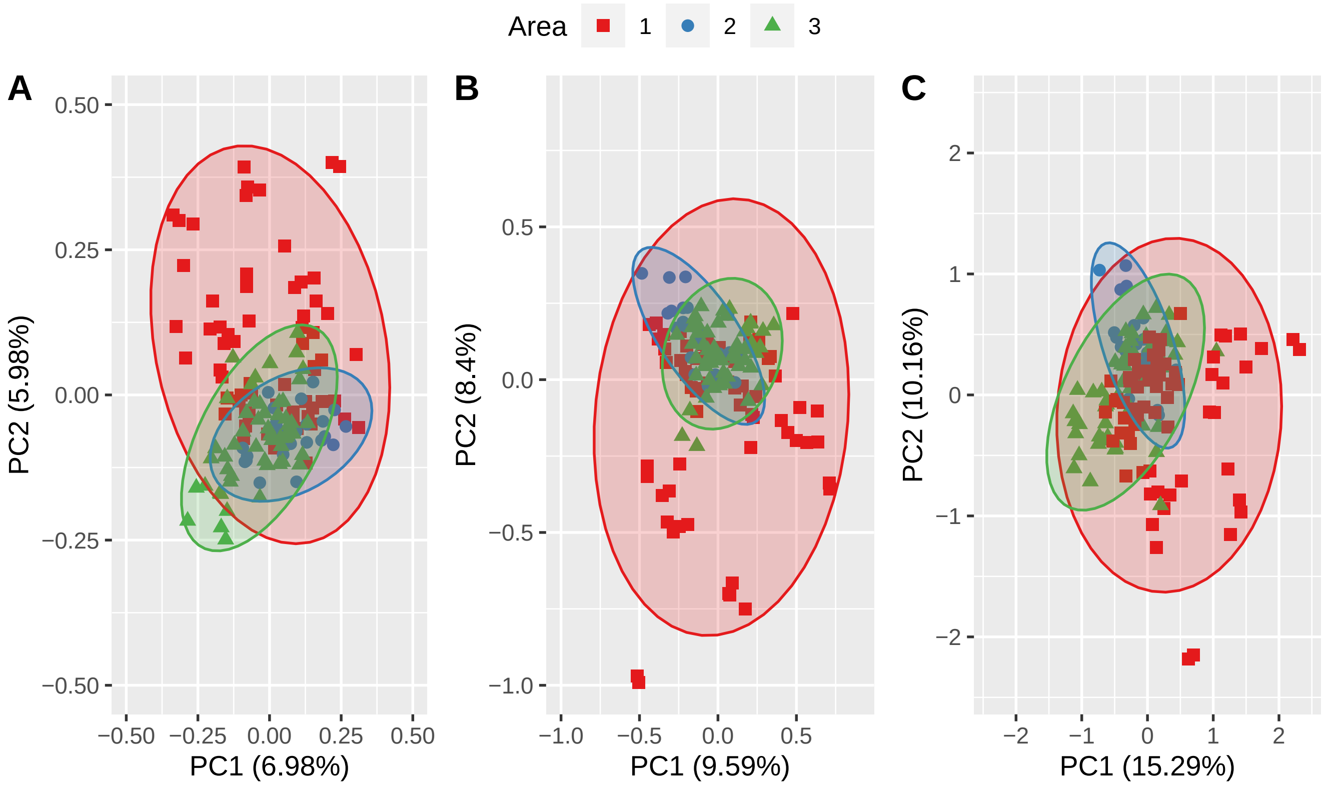

Figure S3. Principal component analysis plots considering different references for SNP calling: (**A**) *Acrocomia aculeata* transcriptome, (**B**) oil palm (*Elaeis guineensis*) genome, and (**C**) *de novo* sequencing. Genotypes of different groups (areas) have distinct shapes and colours. The ellipses represent the 95% confidence intervals of each group.

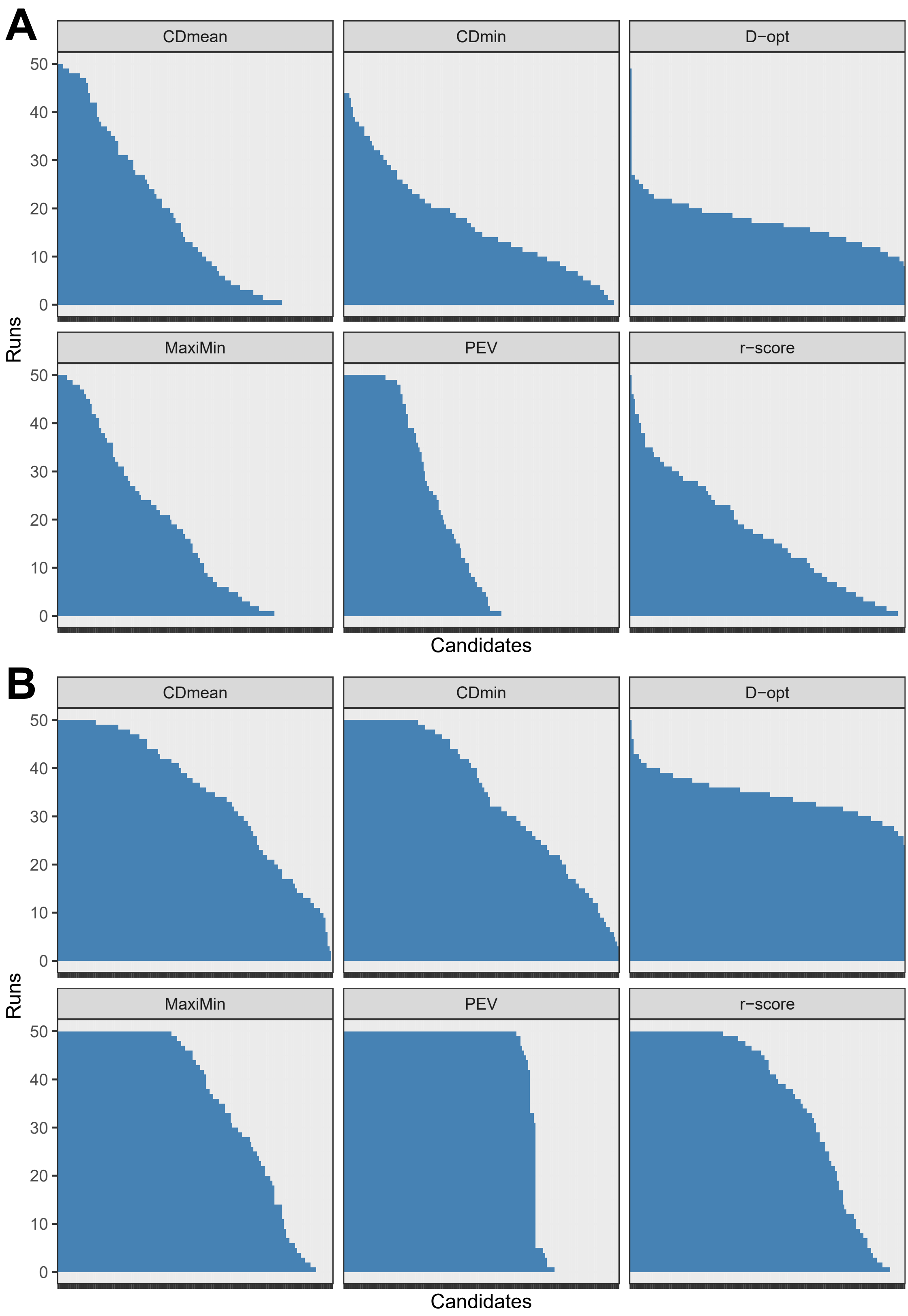

Figure S4. Selection consistency across runs in the untargeted scenario: The bars represent the number of times a given candidate (*x*-axis) was selected across runs (*y*-axis), considering a training set size of 50 (**A**) and 100 (**B**).

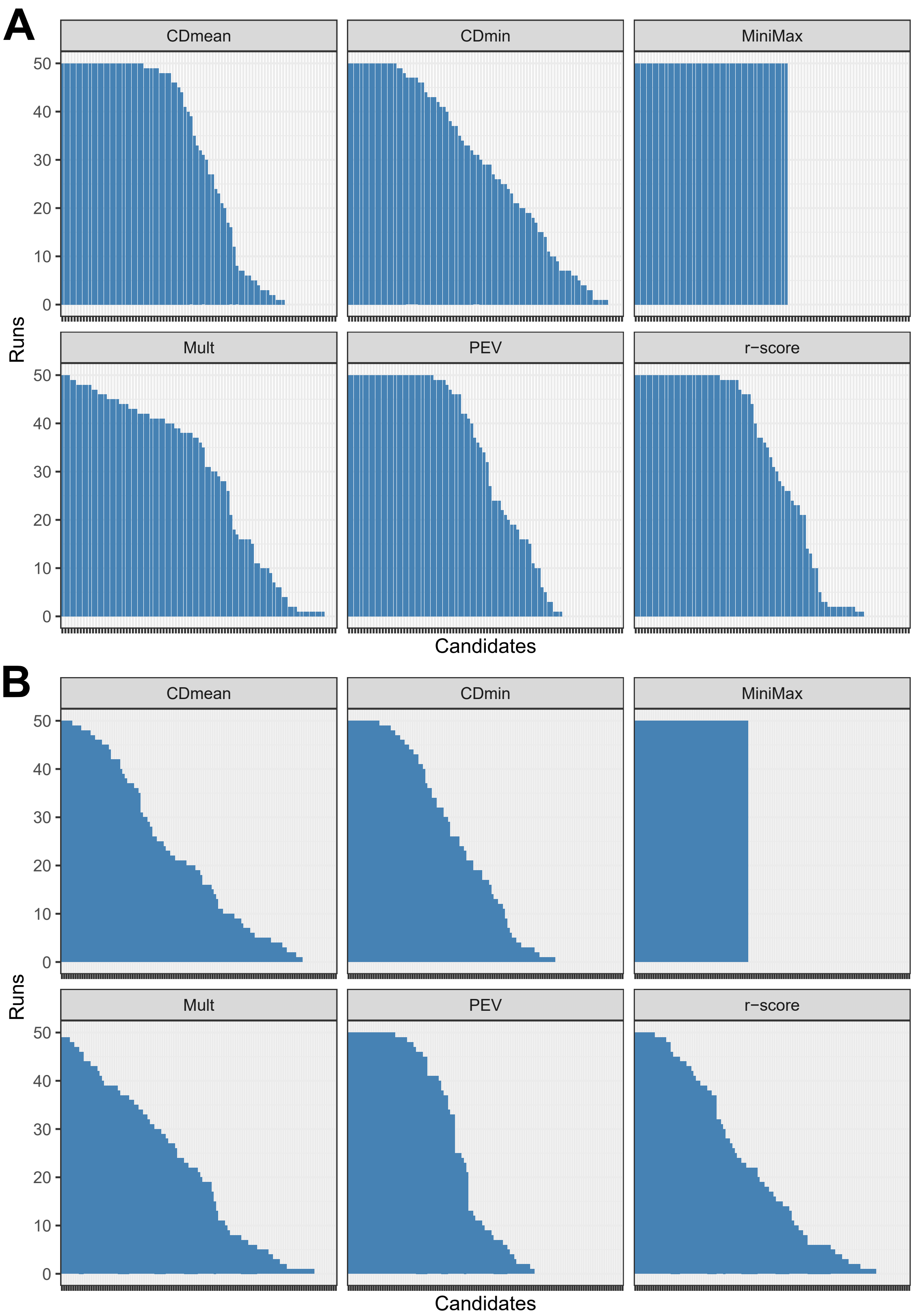

Figure S5. Selection consistency across runs in the targeted scenario: The bars represent the number of times a given candidate (*x*-axis) was selected across runs (*y*-axis), when using groups 1 and 2 to predict group 3 (**A**), and when using groups 1 and 3 to predict group 2 (**B**).
